# Early-Life Diarrhea Disrupts Antioxidant–Immune Homeostasis and Gut Microbiota in Suckling Calves

**DOI:** 10.3390/biology15060450

**Published:** 2026-03-10

**Authors:** Xi Liang, Xueqiang Li, Ningning Mi, Yingga Wu, Jingze Wu, Hui Chen, Dacheng Liu

**Affiliations:** 1 College of Veterinary Medicine, Inner Mongolia Agricultural University, Hohhot 010018, China; 15754882693@163.com (X.L.); lixueqiang2023@163.com (X.L.);; 2 Key Laboratory of Clinical Diagnosis and Treatment of Animal Diseases, Ministry of Agriculture, Hohhot 010018, China; 3 National Center of Technology Innovation for Dairy, Hohhot 010018, China

**Keywords:** calf diarrhea, gut microbiota, oxidative stress, immune function, intestinal barrier

## Abstract

Calf diarrhea is a common early-life disorder that adversely affects growth, oxidative balance, immune function, and intestinal microbiota, thereby compromising health and production performance. This study systematically investigates the effects of naturally occurring diarrhea in 7-day-old suckling calves on oxidative stress, immune responses, intestinal barrier integrity, and gut microbiota structure and function. Fecal scores, serum antioxidant and immune indices, and intestinal permeability markers were measured, and fecal samples were subjected to metagenomic sequencing. Diarrhea-affected calves exhibited higher fecal scores, increased oxidative stress indicated by reduced total antioxidant capacity, elevated lipid peroxidation, and altered antioxidant enzyme activities. Humoral immunity was impaired, inflammatory responses were dysregulated, and intestinal barrier function was disrupted. Gut microbial diversity declined, showing a depletion in health-associated taxa and the enrichment of opportunistic pathogens. Correlation analyses revealed that pathogenic bacteria abundance positively associated with diarrhea severity, oxidative stress, inflammation, and barrier disruption, while beneficial genera correlated with antioxidant and immune function. Functional profiling indicated a microbial shift from amino acid metabolism and antioxidant homeostasis toward carbohydrate and energy metabolism under diarrheic conditions. These findings highlight the pivotal role of gut microbiota dysbiosis in diarrhea pathogenesis and provide a foundation for developing microbiome-targeted interventions to improve calf health.

## Simple Summary

Calf diarrhea is one of the most common diseases affecting young calves and can lead to growth retardation, increased treatment costs, and even mortality. However, the biological mechanisms underlying diarrhea, particularly the role of gut microbiota, are not fully understood. In this study, we compared healthy calves and calves with diarrhea to investigate changes in fecal characteristics, antioxidant status, immune function, and gut microbial composition and function. We found that diarrhea was associated with increased fecal water content, oxidative stress, immune imbalance, and disruption of intestinal barrier function. At the microbial level, diarrheic calves showed reduced microbial diversity, enrichment of opportunistic pathogens, and alterations in metabolic functions. These findings suggest that gut microbiota imbalance plays a key role in the development of diarrhea and its related physiological disorders. Our study provides new insights into the microbial mechanisms of calf diarrhea and may contribute to the development of nutritional or microbiome-based strategies to improve calf health.

## 1. Introduction

Calf diarrhea is one of the most common and severe diseases affecting suckling calves. It not only markedly increases morbidity and mortality but also impairs growth and development, reduces feed efficiency, and exerts long-term adverse effects on subsequent production performance and lifelong health [1,2]. Despite continuous improvements in calf management practices and disease prevention strategies in recent years, diarrhea remains a major constraint to healthy calf rearing and the sustainable development of the dairy industry [3]. Therefore, systematically elucidating the pathogenesis of calf diarrhea is of substantial theoretical and practical significance for the development of effective prevention and control strategies.

Current evidence suggests that calf diarrhea arises from the combined effects of multiple factors, including pathogenic infections, inappropriate feeding management, nutritional imbalance, and immaturity of the immune system [4]. Following the onset of diarrhea, calves frequently exhibit pronounced oxidative stress and immune dysfunction, characterized by reduced antioxidant defense capacity, dysregulated inflammatory responses, and compromised intestinal barrier integrity [5]. These physiological disturbances not only aggravate diarrheal symptoms but may also exacerbate tissue injury through a self-perpetuating cycle of “inflammation–oxidative stress–barrier impairment” [6]. However, the upstream regulatory mechanisms driving these systemic alterations remain incompletely understood.

As a central hub linking host nutrient metabolism, immune regulation, and intestinal barrier function, the gut microbiota plays a critical role in maintaining intestinal homeostasis and protecting the host against external insults. Accumulating evidence indicates that gut microbiota dysbiosis is closely associated with the occurrence and progression of calf diarrhea [7,8]. Diarrheic calves typically exhibit reduced microbial diversity, depletion of homeostasis-associated commensal bacteria, and enrichment of opportunistic pathogens [7]. These microbial alterations may contribute to diarrhea-related pathophysiology by modulating microbial metabolite production, triggering inflammatory responses, and disrupting intestinal barrier function [9]. Nevertheless, existing studies on gut microbial alterations in diarrheic calves have largely focused on descriptive analyses of community composition based on 16S rRNA gene sequencing, whereas comprehensive investigations into microbial functional potential and its associations with host oxidative stress and immune function remain limited.

Metagenomic sequencing provides a powerful approach for comprehensively characterizing gut microbiota composition at species-level resolution and elucidating microbial functional capacity through in-depth functional annotation, thereby enabling detailed exploration of complex host–microbiota interactions [10]. Integrating microbial structural characteristics, functional profiles, and host phenotypic indicators facilitates a holistic understanding of diarrhea-associated gut dysbiosis and its underlying mechanisms [6]. However, systematic studies addressing gut microbial functional reprogramming and its relationships with oxidative stress, immune dysfunction, and intestinal barrier damage in early-stage diarrheic calves are still scarce.

Therefore, the present study comprehensively evaluates the effects of diarrhea on fecal scores, antioxidant capacity, immune function, and intestinal barrier-related parameters in healthy and diarrheic calves. Metagenomic sequencing was applied to systematically investigate changes in gut microbial diversity, community structure, microbial interaction networks, and functional characteristics under diarrheic conditions. By further analyzing the correlations between gut microbiota alterations and host phenotypic indicators, this study aims to elucidate the characteristics and potential mechanisms of diarrhea-associated gut dysbiosis. The findings provide a theoretical basis for advancing the understanding of calf diarrhea pathogenesis and for developing future nutritional regulation or microbiome-targeted intervention strategies to improve the health status of diarrheic calves.

## 2. Materials and Methods

### 2.1. Experimental Design

The experiment was conducted at a large-scale commercial dairy farm located in Suihua City, Heilongjiang Province, China. The experimental protocol was reviewed and approved by the Animal Welfare and Ethics Committee of Inner Mongolia Agricultural University (Approval No.: NND2023123). All experimental procedures were performed in strict accordance with the National Research Council guidelines for the care and use of laboratory animals (2022-6-10/SYXK 2022-0031).

A total of twelve 7-day-old Holstein calves were enrolled in this study. All calves were obtained from the same calving batch and exhibited similar initial body weights (38.5 ± 0.67 kg). Based on clinical fecal characteristics and the occurrence of diarrhea, calves were allocated to either a Healthy group (*n* = 6) or a Diarrheic group (*n* = 6). Calves in the Healthy group maintained normal fecal consistency and showed no signs of diarrhea throughout the observation period, whereas calves in the Diarrheic group were naturally occurring cases of diarrhea. Prior to group allocation, all calves underwent routine clinical examinations conducted by farm veterinarians, including rectal temperature measurement and general health assessment. No laboratory-based diagnostic tests (e.g., pathogen identification or blood biochemical analysis) were performed before enrollment.

This study was designed as a cross-sectional observational comparison at a single time point (7 days of age) rather than an etiological investigation. Pathogen-specific diagnostics (e.g., viral PCR, bacterial culture, or parasitological examination) were not conducted; therefore, diarrhea classification was based solely on standardized clinical fecal scoring criteria.

All calves were subjected to identical feeding management practices and environmental conditions from birth to 7 days of age, including colostrum feeding, milk replacer feeding regimens, and hygienic management protocols. Prior to sample collection, calves were housed individually in separate calf pens to prevent cross-contamination and minimize environmental interference with experimental outcomes.

Diarrhea was evaluated using a standardized fecal scoring system based on a 5-point scale, where 1 indicated normal, well-formed feces and 5 indicated watery diarrhea. Diarrhea was diagnosed when calves exhibited a fecal score ≥ 3 in two consecutive assessments within a 24 h period. To ensure sample integrity and data reliability, none of the calves received antibiotics, feed additives, or any pharmacological treatment prior to sample collection. As this study was conducted as a single time-point sampling at 7 days of age, no post-sampling follow-up was performed.

The sample size (*n* = 6 per group) was determined based on animal availability within the same calving batch and logistical constraints. Given the exploratory nature of this study, no a priori power calculation was performed; therefore, the statistical analyses should be interpreted as exploratory and hypothesis-generating.

### 2.2. Sample Collection

#### 2.2.1. Serum Sample Collection

Approximately 10 mL of blood was collected from the jugular vein of each calf using vacuum blood collection tubes containing a coagulation activator. Blood samples were allowed to clot at room temperature and then centrifuged at 3000 rpm for 10 min to obtain serum. The separated serum was aliquoted into sterile 2 mL enzyme-free cryovials, appropriately labeled, and immediately stored at −80 °C until analysis.

Serum samples were used to determine the concentrations of immunoglobulin G (IgG), immunoglobulin A (IgA), interleukin-2 (IL-2), interleukin-4 (IL-4), interleukin-10 (IL-10), tumor necrosis factor-α (TNF-α), diamine oxidase (DAO), endotoxin (ET), total antioxidant capacity (T-AOC), superoxide dismutase (SOD), glutathione peroxidase (GSH-Px), and malondialdehyde (MDA).

#### 2.2.2. Fecal Sample Collection

Fresh fecal samples were collected directly from the rectum of each calf using sterile disposable gloves. Approximately 10 g of feces was obtained and immediately transferred into sterile centrifuge tubes to avoid environmental contamination. Samples were rapidly flash-frozen in liquid nitrogen and subsequently stored at −80 °C until DNA extraction and metagenomic sequencing analysis.

### 2.3. Index Measurement and Methods

#### 2.3.1. Fecal Scoring

Fecal consistency was evaluated according to the method described by Larson et al. [11]. The detailed scoring criteria are presented in Table 1. A fecal score ≥ 3 was considered indicative of diarrhea. All fecal scoring assessments were independently performed by a licensed veterinarian at the farm using standardized criteria to minimize observer bias.

#### 2.3.2. Determination of Antioxidant Capacity Indexes

Serum antioxidant indices, including total antioxidant capacity (T-AOC), total superoxide dismutase (T-SOD), glutathione peroxidase (GSH-Px), and malondialdehyde (MDA), were measured using commercially available assay kits. Detailed information regarding the assay methods and kit specifications is provided in Table S1. All kits were purchased from Ruixin Biological (Quanzhou, China), and assays were conducted strictly in accordance with the manufacturer’s instructions.

#### 2.3.3. Determination of Immune Function Indexes

Serum immune-related parameters, including IgG, IgA, IL-2, IL-4, IL-10, TNF-α, ET, and DAO, were quantified using commercially available assay kits. Detailed assay principles and kit information are summarized in Table S2. All kits were obtained from Ruixin Biological (Quanzhou, China), and measurements were performed according to the manufacturer’s protocols.

#### 2.3.4. Metagenomic Sequencing and Bioinformatic Analysis

Total genomic DNA was extracted from faecal samples using the E.Z.N.A.^®^ Soil DNA Kit (Omega Bio-tek, Norcross, GA, USA) according to the manufacturer’s protocol. The soil DNA extraction kit was selected due to its strong mechanical lysis efficiency and inhibitor removal capacity, which are advantageous for complex fecal matrices rich in polysaccharides and PCR inhibitors. However, differential cell lysis efficiency may introduce taxonomic bias, particularly affecting Gram-positive bacteria, and this potential limitation should be considered when interpreting compositional differences.

DNA integrity was evaluated by 1% agarose gel electrophoresis, and DNA concentration and purity were assessed spectrophotometrically. Purified DNA was subsequently fragmented to an average size of approximately 350 bp using a Covaris M220 ultrasonicator (Covaris Inc., Woburn, MA, USA), and paired-end sequencing libraries were constructed using the NEXTFLEX Rapid DNA-Seq Kit (Bioo Scientific, Austin, TX, USA).

Metagenomic sequencing was performed on the Illumina NovaSeq™ X Plus platform (Illumina, San Diego, CA, USA), with sequencing services provided by Shanghai Majorbio Bio-Pharm Technology Co., Ltd. (Shanghai, China). On average, each sample generated approximately 10 Gb of raw data (paired-end 150 bp reads). Raw reads were subjected to quality filtering using fastp software (v0.23.2) to remove adapter sequences, reads with >10% ambiguous bases (N), and low-quality reads (Q-score < 20 over more than 50% of bases). After quality control, an average of 80 million clean reads per sample was retained for downstream analyses.

Raw sequencing reads were initially aligned to the host genome using Burrows–Wheeler Aligner (BWA, v0.7.17) [12] to remove host-derived sequences. Host-derived reads were filtered by mapping to the Bos taurus reference genome (ARS-UCD1.2), and only non-host high-quality reads were retained. High-quality, non-host reads were then assembled de novo using MEGAHIT software (v1.1.2) [13], and contigs with a minimum length of 300 bp were retained for downstream analyses.

Open reading frames (ORFs) were predicted from the assembled contigs using Prodigal software (v2.6.3), and gene sequences with lengths ≥ 100 bp were selected for further analysis. The predicted nucleotide sequences were translated into their corresponding amino acid sequences. All predicted amino acid sequences from each sample were clustered using CD-HIT software (v4.7) [14] with a sequence identity threshold of 90% and a coverage threshold of 90%. The longest sequence from each cluster was selected to generate a non-redundant gene catalogue.

High-quality reads from each sample were mapped back to the non-redundant gene catalogue using SOAPaligner software (v2.21) [15] with a sequence identity threshold of 95% to quantify gene abundance. For taxonomic annotation, amino acid sequences from the non-redundant gene set were aligned against the NCBI non-redundant (NR) protein database using DIAMOND software (v2.0.13) [16] in BLASTP (v2.13.0) mode with an e-value cutoff of 1 × 10^−5^. Taxonomic assignments were determined based on the NR annotation results, and the relative abundance of each microbial species was calculated as the sum of the abundances of its corresponding genes.

Alpha diversity indices were calculated to evaluate microbial community richness and diversity at the species level. The Chao1 indices were used to estimate species richness, whereas the Shannon and Simpson indices were used to assess community diversity. These indices were calculated based on species abundance matrices using QIIME2 (v2022.2) and R (v4.2.1) software.

Functional annotation of predicted genes was performed against the KEGG database (release version 20241007) using DIAMOND (v2.0.15) with an e-value threshold of 1 × 10^−5^. Pathway abundances were calculated by summing the abundances of genes assigned to corresponding KEGG Orthology (KO) entries.

Raw metagenomic sequencing data have been deposited in the NCBI Sequence Read Archive (SRA) under BioProject accession number PRJNA1428989, in accordance with current data-sharing standards.

### 2.4. Statistical Analysis

All data were organized using Microsoft Excel 2010 and analyzed with IBM SPSS Statistics 22.0. Differences between groups were evaluated using independent samples *t*-tests. Results are expressed as mean ± SD. Differences were considered statistically significant at *p* < 0.05 and highly significant at *p* < 0.01.

Prior to parametric testing, data normality was assessed using the Shapiro–Wilk test, and homogeneity of variance was evaluated using Levene’s test. When assumptions were not met, non-parametric Mann–Whitney U tests were applied.

Spearman’s rank correlation analysis was used to assess associations between gut microbiota and serum antioxidant and immune parameters. In addition, Pearson correlation analysis was performed to evaluate co-occurrence relationships among dominant microbial taxa based on their relative abundances. Correlation matrices were visualized using heatmaps, and only correlations with *p* < 0.05 were considered statistically significant.

Given the limited sample size, correlation and co-occurrence network analyses should be interpreted as exploratory. Network topology metrics were not used to infer causality but to describe potential association patterns among taxa.

For metagenomic differential analyses, *p*-values were adjusted using the Benjamini–Hochberg false discovery rate (FDR) correction, and adjusted *p*-values (q-values) < 0.05 were considered statistically significant.

## 3. Results

### 3.1. Effect of Diarrhea on FECAL Scoring in Calves

Fecal scoring is a reliable indicator reflecting the occurrence and severity of diarrhea in calves. Compared with healthy calves, the diarrheic group exhibited a significantly higher fecal score (Table 2; *p* < 0.01), indicating a marked deterioration in fecal consistency following the onset of diarrhea.

### 3.2. Effect of Diarrhea on Antioxidant Capacity in Calves

To evaluate the impact of diarrhea on systemic oxidative status, serum antioxidant-related indices were compared between groups (Table 3). Total antioxidant capacity (T-AOC) was significantly reduced in the diarrheic group (*p* < 0.01), indicating impaired overall antioxidant defense. In contrast, superoxide dismutase (SOD) and glutathione peroxidase (GSH-Px) activities were significantly elevated (*p* < 0.05), suggesting a compensatory enzymatic response to increased oxidative stress.

Furthermore, serum malondialdehyde (MDA) levels were markedly higher in diarrheic calves than in healthy controls (*p* < 0.01), indicating enhanced lipid peroxidation and aggravated oxidative damage.

Collectively, these findings demonstrate that diarrhea significantly disrupts redox homeostasis in calves, characterized by decreased total antioxidant capacity and increased oxidative damage. Although enzymatic antioxidant activities were upregulated, this compensatory response appeared insufficient to counteract diarrhea-induced oxidative stress.

### 3.3. Effects of Diarrhea on Immune Function in Calves

Serum immunoglobulins, cytokines, and intestinal permeability-related indices were compared between groups (Table 4). Serum IgG concentrations were significantly reduced in diarrheic calves (*p* < 0.01), whereas IgA levels did not differ significantly, indicating selective impairment of humoral immune function.

Regarding cytokines, pro-inflammatory TNF-α levels were significantly elevated (*p* < 0.01) in the diarrheic group. Anti-inflammatory IL-10 levels were also significantly increased (*p* < 0.05), whereas IL-2 and IL-4 showed no significant differences. These results suggest that diarrhea induces a pronounced inflammatory response accompanied by compensatory immunoregulatory activity.

Serum diamine oxidase (DAO) and endotoxin (ET) concentrations were significantly higher in diarrheic calves (*p* < 0.05), indicating compromised intestinal barrier integrity and increased intestinal permeability.

Overall, diarrhea markedly impaired humoral immune function, induced inflammatory dysregulation, and exacerbated intestinal barrier damage, highlighting immune dysfunction and barrier disruption as key pathological features of diarrheic calves.

### 3.4. Effects of Diarrhea on Intestinal Microbial Diversity in Calves

**Figure 1:**
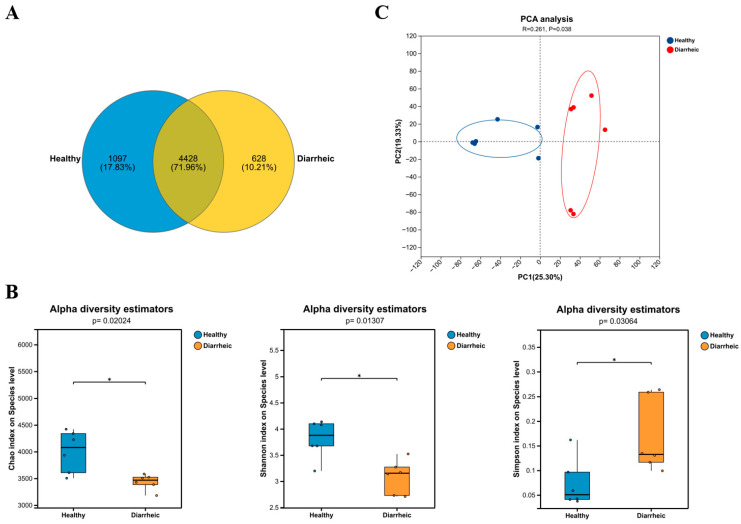
Effects of diarrhea on gut microbial diversity and community structure in calves. (**A**) Venn diagram showing shared and unique amplicon sequence variants (ASVs) between healthy and diarrheic calves. (**B**) Alpha diversity indices (Shannon, Chao1, and Simpson) of gut microbiota in healthy and diarrheic groups. (**C**) Principal component analysis (PCA) based on Bray–Curtis distance illustrating differences in overall microbial community structure between groups. Data are presented as mean ± SD. Differences between groups were analyzed using independent samples *t*-tests (alpha diversity) and ANOSIM (community structure). *p* < 0.05 was considered statistically significant. * *p* < 0.05. *n* = 6.

Metagenomic sequencing of fecal samples was performed using the Illumina NovaSeq™ X Plus platform (Illumina, San Diego, CA, USA). A total of 6153 microbial taxa were detected across all samples. Among them, 4428 taxa (71.96%) were shared between healthy and diarrheic calves (Figure 1A). The healthy and diarrheic groups harbored 1097 (17.83%) and 628 (10.21%) unique taxa, respectively.

Alpha diversity analysis (Figure 1B) revealed that the Shannon and Chao indices were significantly reduced in the diarrheic group (*p* < 0.05), whereas the Simpson index was significantly increased (*p* < 0.05). These results indicate decreased microbial richness and diversity accompanied by increased dominance, suggesting substantial disruption of gut microbial homeostasis.

Principal component analysis (PCA) demonstrated a clear separation between healthy and diarrheic samples along the first two principal components (Figure 1C; *p* < 0.05). Intragroup clustering was relatively compact, indicating good within-group consistency. ANOSIM analysis further confirmed significant differences in community structure between groups (*p* < 0.05).

These findings indicate that diarrhea significantly alters gut microbial diversity and overall community structure in calves.

### 3.5. Effects of Diarrhea on Calf Gut Microbiome Composition

Metagenomic annotation identified 91 phyla, 137 classes, 1484 genera, and 6153 species in total.

**Figure 2:**
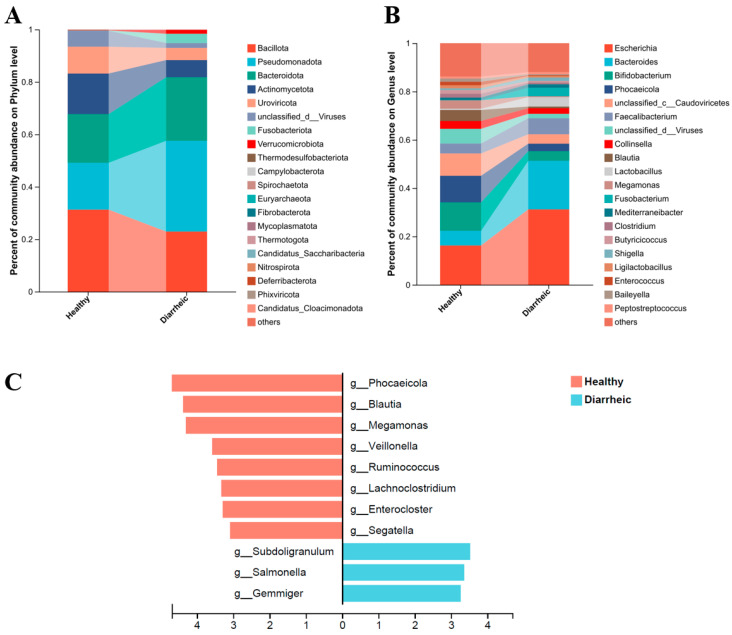
Diarrhea alters gut microbial taxonomic composition in calves. (**A**) Relative abundance of dominant bacterial phyla in healthy and diarrheic calves. (**B**) Relative abundance of the top 20 most abundant genera between groups. (**C**) LEfSe analysis identifying differentially enriched taxa (LDA ≥ 3.0, *p* < 0.05). Taxa enriched in healthy calves are shown in red, and taxa enriched in diarrheic calves are shown in blue. Data are presented as mean relative abundance. Statistical significance between groups was determined using the Wilcoxon rank-sum test. *n* = 6.

At the phylum level (Figure 2A), dominant taxa in both groups included *Bacillota*, *Pseudomonadota*, *Bacteroidota*, and *Actinobacteriota*. The relative abundance of *Bacillota* was significantly lower in diarrheic calves (*p* < 0.05), whereas *Pseudomonadota* showed an increasing trend without reaching statistical significance.

At the genus level (Figure 2B), the top 20 most abundant genera were compared. Dominant genera included *Escherichia*, *Bacteroides*, and *Bifidobacterium*, with significant differences in relative abundance between health statuses.

LEfSe analysis (LDA ≥ 3, *p* < 0.05) revealed significant compositional differences (Figure 2C). Genera enriched in healthy calves included *Phocaeicola*, *Blautia*, *Megamonas*, *Veillonellales*, *Ruminococcus*, *Lachnoclostridium*, *Enterocloster*, and *Segatella*. In contrast, *Subdoligranulum*, *Salmonella*, and *Gemmiger* were significantly enriched in diarrheic calves.

### 3.6. Correlation Analysis

#### 3.6.1. Microbiota–Host Phenotype Correlation

**Figure 3:**
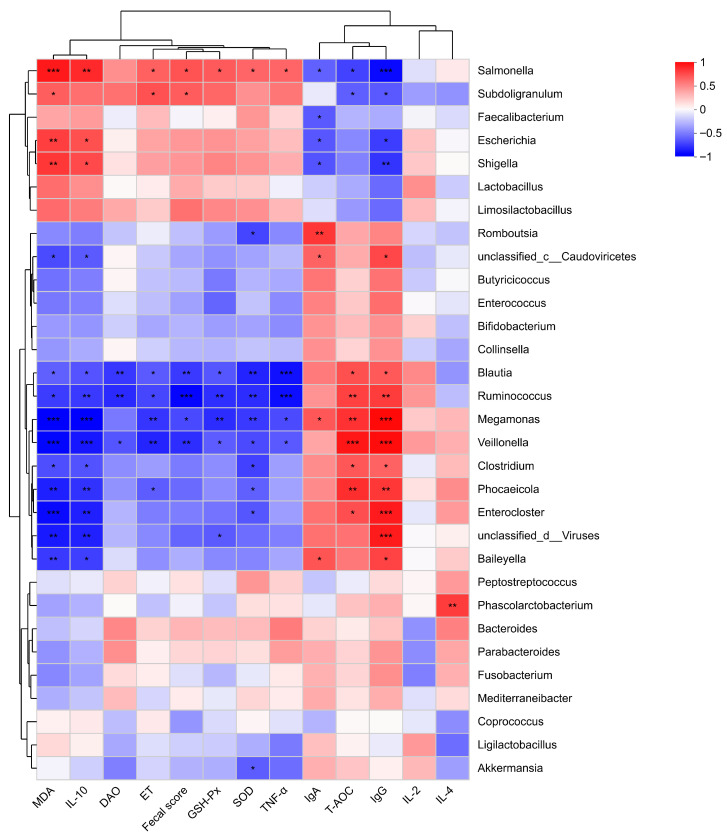
Correlation analysis between gut microbiota and host phenotypic parameters. Spearman correlation heatmap showing associations between representative genera and host parameters, including fecal score, oxidative stress markers (MDA, GSH-Px, SOD, T-AOC), inflammatory cytokines (IL-10, TNF-α), immune indicators (IgG, IgA), and intestinal barrier markers (DAO, ET). Red indicates positive correlations and blue indicates negative correlations. The intensity of color corresponds to the strength of correlation. * *p* < 0.05, ** *p* < 0.01, *** *p* < 0.001. *n* = 6.

Spearman correlation analysis (Figure 3; Additional File S1) revealed that opportunistic pathogens were positively associated with diarrhea severity and inflammatory/oxidative markers.

*Salmonella* showed positive correlations with fecal score, MDA, IL-10, ET, GSH-Px, SOD, and TNF-α, and negative correlations with IgG, IgA, and T-AOC. *Subdoligranulum* positively correlated with fecal score, MDA, and ET, but negatively with IgG and T-AOC. *Escherichia* and *Shigella* were positively associated with MDA and IL-10 and negatively associated with IgG and IgA.

Conversely, beneficial genera enriched in healthy calves—including *Blautia*, *Ruminococcus*, *Megamonas*, and *Veillonella*—were negatively correlated with fecal score, MDA, IL-10, DAO, ET, GSH-Px, SOD, and TNF-α, and positively correlated with IgG and/or T-AOC. Similar patterns were observed for Clostridium, *Phocaeicola*, *Enterocloster*, and *Baileyella*.

These results indicate that enrichment of opportunistic pathogens is associated with exacerbated oxidative stress, inflammation, and barrier dysfunction, whereas depletion of beneficial bacteria may weaken antioxidant and immune defenses.

#### 3.6.2. Genus-Level Correlation Network Analysis

**Figure 4:**
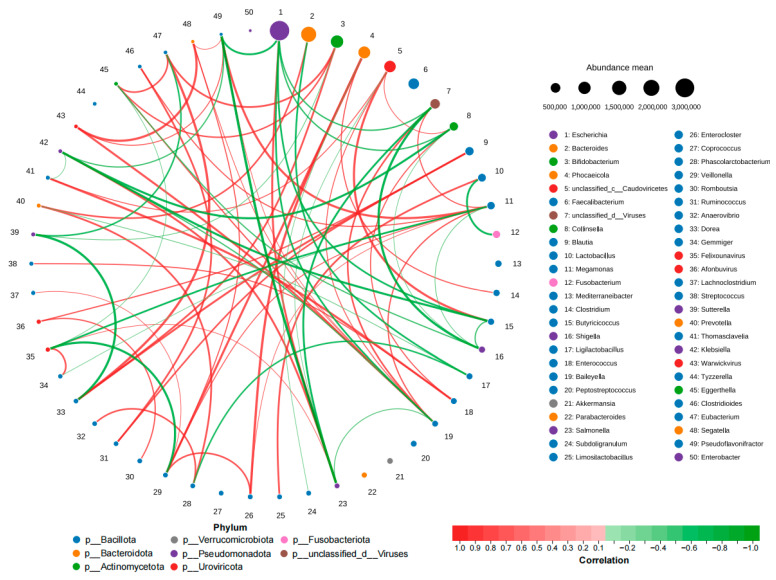
Genus-level ecological correlation network of the calf gut microbiota. Pearson correlation-based network analysis illustrating ecological interactions among bacterial genera. Nodes represent genera, and edges represent significant correlations (|r| > 0.8, *p* < 0.05). Red edges indicate positive correlations, and blue edges indicate negative correlations. The thickness of edges corresponds to correlation strength. *n* = 6.

A Pearson correlation-based network analysis was conducted to evaluate ecological interactions among genera (Figure 4).

Strong positive correlations were observed among opportunistic pathogens, including Escherichia–Salmonella (r = 0.87) and Salmonella–Klebsiella (r = 0.87), suggesting synergistic proliferation under diarrheic conditions.

In contrast, beneficial bacteria exhibited antagonistic relationships with pathogens. For example, Lactobacillus showed a strong negative correlation with Fusobacterium (r = −0.87), indicating reduced probiotic-mediated suppression of inflammation-associated taxa.

Positive correlations were also observed among genera associated with energy metabolism and intestinal homeostasis, including Ruminococcus–Clostridioides (r = 0.86), Megamonas–Clostridioides (r = 0.83), Collinsella–Enterococcus (r = 0.83), and Enterococcus–Streptococcus (r = 0.83).

Overall, diarrhea induced not only compositional shifts but also ecological network restructuring, characterized by synergistic expansion of opportunistic pathogens and weakened antagonistic interactions of beneficial bacteria.

### 3.7. Differential Analysis of Gut Microbiota Functional Characteristics

The foregoing results demonstrate that diarrhea not only alters gut microbial diversity and taxonomic composition in calves but also substantially reshapes microbial interaction networks. Considering that structural alterations in microbial communities are frequently accompanied by changes in functional capacity, further analyses were conducted to characterize functional reprogramming of the gut microbiota under diarrheic conditions.

Based on metagenomic sequencing data, comparative KEGG pathway analysis was performed to evaluate functional differences between healthy and diarrheic calves and to determine the potential functional consequences of diarrhea-associated gut dysbiosis.

**Figure 5:**
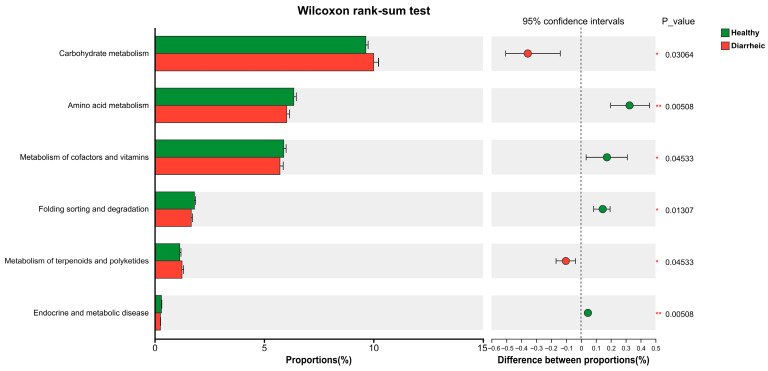
Functional differences of gut microbiota between healthy and diarrheic calves based on KEGG pathway analysis. Comparative analysis of KEGG Level 2 functional categories between groups. Bars represent the relative abundance of annotated pathways. Significant differences between healthy and diarrheic calves were determined using the Wilcoxon rank-sum test. * *p* < 0.05, ** *p* < 0.01. *n* = 6.

KEGG Level 2 functional annotation (Figure 5; Additional File S2) revealed significant differences (*p* < 0.05) in multiple metabolic and cellular process-related pathways between groups. Pathways associated with carbohydrate metabolism and the metabolism of terpenoids and polyketides were significantly enriched in the diarrheic group. In contrast, the healthy group exhibited significantly higher relative abundances in pathways related to amino acid metabolism, metabolism of cofactors and vitamins, protein folding, sorting and degradation, and endocrine and metabolic disease-associated pathways (*p* < 0.05).

To further refine these functional alterations, KEGG Level 3 pathway analysis was conducted (Additional File S3). Compared with the diarrheic group, healthy calves showed significantly greater functional representation in pathways involved in amino acid biosynthesis and antioxidant-related metabolism, including biosynthesis of amino acids, branched-chain amino acid biosynthesis, pantothenate and CoA biosynthesis, and tryptophan and tyrosine metabolism (*p* < 0.05).

Conversely, pathways related to carbohydrate digestion and absorption and energy metabolism were significantly enriched in the diarrheic group, including carbohydrate digestion and absorption, propanoate metabolism, and pyrimidine metabolism (*p* < 0.05). Differential enrichment was also observed in signaling pathways associated with metabolic regulation and oxidative stress, such as the PPAR and FoxO signaling pathways.

Collectively, these findings indicate that under diarrheic conditions, the functional profile of the calf gut microbiome shifts from a health-associated pattern characterized by amino acid metabolism and antioxidant homeostasis toward a profile marked by enhanced carbohydrate utilization and energy metabolism. This functional shift is consistent with the observed structural alterations in microbial communities and their correlations with fecal scores, antioxidant status, and immune parameters.

## 4. Discussion

This study systematically compares fecal scores, antioxidant capacity, immune function, gut microbial structure, and functional characteristics between healthy calves and diarrheic calves at 7 days of age. From an integrated “phenotype–microbiota–function” perspective, we aimed to elucidate the potential mechanisms underlying diarrhea-associated microbial dysbiosis. The results demonstrate that diarrheic calves exhibited significantly elevated fecal scores, accompanied by impaired antioxidant defenses and disrupted immune homeostasis. Moreover, gut microbial diversity, community composition, and functional metabolic profiles were comprehensively reshaped. Correlation analyses further revealed significant associations between multiple differentially abundant bacterial genera and fecal scores, antioxidant indicators, and immune parameters. However, given the relatively limited sample size of the present study, these associations should be interpreted cautiously and regarded as hypothesis-generating rather than definitive evidence of causality. Collectively, these findings reinforce the close linkage between intestinal microbial imbalance and host health impairment during diarrhea, providing novel microbiological evidence for understanding the pathogenesis of calf diarrhea.

It should also be emphasized that this study was designed as an observational comparative analysis. Therefore, causal inferences regarding microbiota-driven mechanisms cannot be definitively established, and the conclusions should be interpreted within this context.

### 4.1. Comprehensive Effects of Diarrhea on Fecal Characteristics, Oxidative Stress Status, and Immune Function in Calves

Fecal scoring serves as a direct and reliable indicator of diarrhea occurrence and severity in calves. In the present study, fecal scores were significantly higher in the diarrhea group compared to the healthy group, indicating markedly increased fecal looseness and severe disruption of intestinal digestion, absorption, and water–electrolyte balance following diarrhea onset. This observation is consistent with previous studies reporting intestinal dysfunction in diarrheic calves, in which altered fecal consistency represents one of the most direct clinical manifestations [17,18].

Beyond intestinal dysfunction, diarrhea profoundly disturbed systemic oxidative–antioxidative balance. Compared with healthy calves, diarrheic calves exhibited significantly decreased serum T-AOC levels and significantly elevated MDA concentrations, indicating enhanced oxidative stress and aggravated lipid peroxidation damage. Although SOD and GSH-Px activities showed an increasing trend in the diarrhea group—possibly reflecting compensatory antioxidant responses—the overall reduction in total antioxidant capacity suggests that these mechanisms were insufficient to neutralize excessive reactive oxygen species. The increased activities of SOD and GSH-Px in diarrheic calves may represent an adaptive response to elevated oxidative stress during intestinal inflammation. Excessive production of reactive oxygen species (ROS) can activate endogenous antioxidant defense systems, particularly through the Kelch-like ECH-associated protein 1 (Keap1)/Nuclear factor erythroid 2–related factor 2 (Nrf2) signaling pathway. Activation of Nrf2 promotes the transcription of antioxidant enzyme genes, including SOD and GSH-Px, thereby enhancing enzymatic activities in an attempt to restore redox homeostasis. However, when oxidative stress exceeds the buffering capacity of these defense systems, overall antioxidant status may remain compromised. Previous studies have demonstrated that diarrhea-associated inflammation, pathogen infection, and impaired nutrient absorption can all promote oxidative stress. In turn, oxidative stress further damages intestinal epithelial cells, exacerbates barrier dysfunction, and perpetuates a vicious cycle of intestinal injury [19,20].

Regarding immune function, serum IgG levels were significantly reduced in diarrheic calves, indicating suppression of humoral immune defense [21]. In contrast, IgA levels showed no significant difference, which may be attributable to the immaturity of the neonatal immune system or the complex regulatory mechanisms governing mucosal immunity [22]. Concurrently, diarrhea markedly elevated the pro-inflammatory cytokine TNF-α, accompanied by increased IL-10 levels, suggesting a state of concurrent inflammatory activation and immune dysregulation during diarrhea [23,24]. Furthermore, the significant elevation of serum DAO and ET levels indicates compromised intestinal barrier integrity and increased permeability, facilitating endotoxin translocation into systemic circulation and thereby amplifying systemic inflammatory responses and immune imbalance [25,26].

Collectively, diarrheic calves exhibited a spectrum of pathological alterations, including abnormal fecal characteristics, heightened oxidative stress, immune dysregulation, and impaired intestinal barrier function. These findings are consistent with previous reports in neonatal calves and confirm that diarrhea is accompanied by multi-system physiological disruption [27]. Importantly, gut microbiota—recognized regulators of barrier integrity, host immunity, and oxidative balance—may contribute substantially to these processes [28,29]. Nevertheless, because pathogen-specific diagnostics were not performed in this study, the precise etiological drivers (e.g., viral, bacterial, or protozoal agents) underlying diarrhea in individual calves remain undetermined. This limitation restricts mechanistic interpretation and prevents attribution of microbial shifts to specific infectious causes.

### 4.2. Structural Features and Potential Mechanisms of Diarrhea-Related Microbial Dysbiosis in Calves

This study reveals multidimensional alterations in calf gut microbiota under diarrhea conditions, encompassing reduced diversity, reshaped community structure, modified microbial interactions, and altered functional potential. These findings provide mechanistic insights into diarrhea-associated physiological dysregulation from a microbiome perspective.

Gut microbial diversity constitutes a fundamental ecological basis for maintaining intestinal homeostasis and resisting pathogen colonization [30]. α-diversity analysis demonstrated significantly reduced Shannon and Chao indices, along with elevated Simpson indices, in diarrheic calves. These findings indicate decreased species richness and evenness and increased dominance concentration within the microbial community [31]. Such patterns are consistent with previous studies in calf diarrhea, weaning stress, and intestinal infection models, which identify reduced microbial diversity as a hallmark of dysbiosis [32]. Decreased diversity weakens microbial functional redundancy and ecological buffering capacity, rendering the intestinal environment more susceptible to opportunistic pathogen colonization and subsequent inflammatory injury [33,34]. PCA and ANOSIM analyses further confirmed that diarrhea significantly reshaped overall microbial structure, indicating systemic rather than incidental microbiome alterations.

At the phylum level, although dominant taxa were shared between groups, diarrheic calves exhibited significantly reduced relative abundance of Firmicutes and a tendency toward increased Proteobacteria. The Firmicutes phylum encompasses numerous short-chain fatty acid (SCFA)-producing bacteria critical for energy metabolism and barrier maintenance. Their depletion is closely associated with impaired intestinal energy supply and heightened inflammation. Conversely, expansion of Proteobacteria is widely regarded as a microbial signature of intestinal inflammation and oxidative stress [35,36,37,38].

Genus-level analysis and LEfSe further delineated diarrhea-associated dysbiosis. Genera enriched in healthy calves, including *Blautia*, *Ruminococcus*, *Megamonas*, and *Phocaeicola*, are strongly linked to SCFA production, barrier maintenance, and immune homeostasis regulation [39,40]. In contrast, genera such as *Salmonella* and *Subdoligranulum* were significantly enriched in the diarrhea group, with certain members possessing confirmed pro-inflammatory or pathogenic properties [41,42]. These findings suggest a transition from a health-associated microbiota characterized by metabolic cooperation and barrier support to a dysbiotic configuration dominated by inflammation-associated and opportunistic taxa.

Spearman correlation analyses provided further evidence linking microbial alterations to host phenotypes. Potentially pathogenic genera including *Salmonella*, *Escherichia*, and *Shigella* showed significant positive correlations with fecal scores, MDA, TNF-α, and ET, and negative correlations with IgG and T-AOC. This suggests that enrichment of these taxa may exacerbate diarrhea-related pathology by enhancing oxidative stress, promoting inflammation, and disrupting intestinal barrier integrity. Conversely, health-associated genera such as *Blautia*, *Ruminococcus*, *Megamonas*, and *Veillonella* demonstrated negative correlations with oxidative stress and inflammatory markers, and positive correlations with antioxidant and immune indices. These results align with previous reports indicating that SCFA-producing bacteria regulate epithelial energy metabolism, suppress inflammatory signaling pathways, and enhance antioxidant defenses, thereby supporting intestinal homeostasis [43,44,45].

However, correlation does not prove causation. These associations indicate coexistence patterns rather than direct mechanistic effects. Experimental validation, such as controlled infection models or microbiota transplantation studies, would be required to confirm causal relationships.

Beyond compositional shifts, microbial interaction networks were also significantly altered. Network analysis revealed strengthened positive correlations among opportunistic pathogens in diarrheic calves, including co-expansion of *Escherichia*, *Salmonella*, and *Klebsiella*, suggesting a permissive intestinal environment for inflammation-associated taxa. Meanwhile, antagonistic relationships between beneficial and pathogenic genera were weakened—for example, the negative correlation between *Lactobacillus* and *Fusobacterium*. This network pattern of “synergistic pathogenicity and weakened antagonism” likely represents a critical ecological mechanism driving the transition from homeostasis to dysbiosis [46,47,48]. Given the modest sample size, ecological network inferences should be interpreted cautiously, as network topology may be sensitive to sample number and sequencing depth.

Metagenomic functional analyses further demonstrated that diarrhea reshaped microbial functional potential. Healthy calves exhibited greater enrichment in amino acid metabolism, cofactor and vitamin metabolism, and antioxidant-related pathways, which are essential for maintaining immune and redox homeostasis [49,50,51]. In contrast, diarrheic calves showed significant enrichment in carbohydrate metabolism, energy metabolism, and selected signaling pathways, suggesting metabolic adaptation to stress conditions. However, such metabolic shifts may promote accumulation of pro-inflammatory metabolites and activation of stress-related signaling cascades, thereby exacerbating barrier dysfunction and systemic oxidative stress [52,53,54]. Importantly, functional annotation was based on metagenomic pathway prediction rather than direct metabolite quantification; therefore, functional shifts represent inferred metabolic potential rather than confirmed biochemical activity.

From a practical perspective, the integrative insights generated in this study provide a theoretical basis for developing nutritional modulation strategies in young calves. Specifically, early-life dietary interventions aimed at enhancing SCFA-producing bacteria, supporting antioxidant capacity (e.g., via functional feed additives such as yeast culture, prebiotics, or plant-derived antioxidants), and stabilizing intestinal barrier integrity may help mitigate diarrhea-associated dysbiosis. Therefore, while the present findings consolidate existing knowledge regarding microbial imbalance and immune impairment, they extend this knowledge by linking microbial functional potential with host oxidative–immune homeostasis, thereby offering actionable targets for precision nutritional regulation in neonatal ruminants.

Regarding methodological considerations, total genomic DNA was extracted using a commercially available soil DNA extraction kit. This kit was selected due to its strong mechanical lysis capacity and inhibitor removal efficiency, which are advantageous for fecal samples rich in complex polysaccharides and PCR inhibitors. Nevertheless, differential lysis efficiency may introduce taxonomic bias, particularly for Gram-positive bacteria. This potential extraction bias should be acknowledged when interpreting compositional differences.

Importantly, these findings should be interpreted as associative rather than causal. The relatively small sample size and absence of pathogen-specific diagnostics limit etiological resolution and mechanistic certainty. Future studies incorporating larger cohorts, longitudinal sampling, and targeted pathogen identification are warranted.

Integrating structural and functional findings, this study delineates microbiome alterations in diarrheic calves. While many of these alterations are consistent with previously reported patterns of microbial imbalance during enteric disease, the present study uniquely integrates oxidative stress indicators, immune parameters, barrier biomarkers, microbial interaction networks, and metagenomic functional profiling within the same early-life cohort. This integrative framework strengthens systems-level understanding of host–microbiota interactions during neonatal diarrhea.

## 5. Conclusions

This study compares oxidative status, immune responses, and gut microbiota composition between healthy and diarrheic calves at 7 days of age. Diarrhea was associated with impaired antioxidant capacity, altered immune parameters, and disruption of intestinal barrier-related indicators. In addition, diarrheic calves exhibited reduced microbial diversity and distinct shifts in microbial community structure. These alterations were significantly correlated with diarrhea severity and host physiological changes. Collectively, our findings highlight the multifactorial impact of early-life diarrhea on calf intestinal health and provide a basis for future strategies aimed at maintaining microbial and physiological stability during the neonatal period.

## Figures and Tables

**Table 1:** Scoring standard of calf feces.

Appearance	Score Standard	Score
Normal	Well-formed and not loose	1
Formed	Soft but formable	2
Unformed	Loose, paste-like	3
Unformed	Watery, easy to spread	4
Unformed	Watery, separated fecal matter, splashing	5

**Table 2:** Fecal scoring criteria for calves.

Items	Healthy Group	Diarrheic Group	*p*-Value
Fecal score/score	1.50 ± 0.55 ^a^	4.17 ± 0.41 ^b^	<0.01

In the same row, values with no letter or the same letter superscripts mean no significant difference (*p* > 0.05), while with different small letter superscripts mean significant difference (*p* < 0.05). The value is the mean ± SD, *n* = 6.

**Table 3:** Effects of diarrhea on antioxidant capacity in calves.

Items	Healthy Group	Diarrheic Group	*p*-Value
T-AOC (ug/mL)	6.60 ± 0.15 ^a^	5.62 ± 0.38 ^b^	<0.01
SOD (U/mL)	44.46 ± 1.89 ^a^	50.46 ± 4.08 ^b^	0.04
GSH-Px (umol/mL)	494.6 ± 22.07 ^a^	541.8 ± 14.42 ^b^	0.01
MDA (nmol/mL)	11.52 ± 1.72 ^a^	15.70 ± 0.53 ^b^	<0.01

In the same row, values with no letter or the same letter superscripts mean no significant difference (*p* > 0.05), while with different small letter superscripts mean significant difference (*p* < 0.05). The value is the mean ± SD, *n* = 6.

**Table 4:** Effects of diarrhea on immune function in calves.

Items	Healthy Group	Diarrheic Group	*p*-Value
IgG (mg/mL)	1.91 ± 0.11 ^a^	1.52 ± 0.12 ^b^	<0.01
IgA (μg/mL)	455.7 ± 42.84	434.2 ± 17.88	0.39
IL-2 (pg/mL)	127.1 ± 15.85	115.1 ± 10.84	0.26
TNF-α (pg/mL)	43.38 ± 1.52 ^a^	51.90 ± 3.44 ^b^	<0.01
IL-10 (pg/mL)	23.97 ± 1.99 ^a^	28.13 ± 1.82 ^b^	0.02
IL-4 (pg/mL)	6.98 ± 0.82	6.94 ± 0.42	0.95
DAO (ng/mL)	44.83 ± 4.09 ^a^	52.93 ± 3.90 ^b^	0.03
ET (pg/mL)	13.72 ± 2.68 ^a^	30.08 ± 3.45 ^b^	<0.01

In the same row, values with no letter or the same letter superscripts mean no significant difference (*p* > 0.05), while with different small letter superscripts mean significant difference (*p* < 0.05). The value is the mean ± SD, *n* = 6.

## Data Availability

The datasets used and analyzed during this study are available from the corresponding author upon reasonable request.

## Supplementary Materials

The following supporting information can be downloaded at https://www.mdpi.com/article/10.3390/biology15060450/s1, Additional File S1: Spearman correlation analysis between differentially abundant genera and fecal scores, antioxidant indices, and immune parameters. Additional File S2: Differential analysis of KEGG Level 2 pathways between healthy and diarrheic calves. Additional File S3: Differential analysis of KEGG Level 3 pathways between healthy and diarrheic calves. Table S1: Serum antioxidant indicator test kits and their product numbers and measurement methods. Table S2: Serum immunological indicator test kit and its product number, testing method.

## Abbreviations

The following abbreviations are used in this manuscript:

ANOSIM	Analysis of similarities
BH	Benjamini–Hochberg
Chao1	Chao1 richness index
CoA	Coenzyme A
DAO	Diamine oxidase
ET	Endotoxin
FDR	False discovery rate
FoxO	Forkhead box O
GSH-Px	Glutathione peroxidase
IgA	Immunoglobulin A
IgG	Immunoglobulin G
IL-2	Interleukin-2
IL-4	Interleukin-4
IL-10	nterleukin-10
KEGG	Kyoto Encyclopedia of Genes and Genomes
LEfSe	Linear discriminant analysis effect size
MDA	Malondialdehyde
NRC	National Research Council
PCA	Principal component analysis
PPAR	Peroxisome proliferator-activated receptor
SCFA	Short-chain fatty acid
SOD	Superoxide dismutase
T-AOC	Total antioxidant capacity
TNF-α	Tumor necrosis factor alpha
