# Supplementary material for: Early-Life Diarrhea Disrupts Antioxidant–Immune Homeostasis and Gut Microbiota in Suckling Calves
Source: Biology (Basel). 2026 Mar 10;15(6):450. doi: 10.3390/biology15060450 (PMC13024218; doi:10.3390/biology15060450)
Supplement: Supplementary file 1 [file biology-15-00450-s001.zip › biology-4179522-supplementary.pdf]

Table S1. Serum antioxidant indicator test kits and their product numbers and measurement methods.

Items	Kit names	method	Product numbers
T-AOC	Total antioxidant capacity assay kit	FRAP	RXFG0297
SOD	Superoxide Dismutase (SOD) assay kit	NBT	RXWB0482
GSH-Px	Glutathione Peroxidase (GSH-Px) assay kit	Visible Color Development Method	RXWB0100
MDA	Malondialdehyde (MDA) assay kit	Micromethod	RXWB0005

Table S2. Serum immunological indicator test kit and its product number, testing method.

Items	Kit names	method	Product numbers
IgG	Immunoglobulin G Assay Kit	ELISA	RX1600039B
IgA	Immunoglobulin A Assay Kit	ELISA	RX1600805B
IL-2	Interleukin -2 Assay Kit	ELISA	RX1600855B
IL-10	Interleukin -10 Assay Kit	ELISA	RX1600646B
IL-4	Interleukin -4 Assay Kit	ELISA	RX1600854B
TNF-α	Bovine Tumor Necrosis Factor-Alpha (TNF-α) Kit	ELISA	RX1600738B
DAO	Bovine Diamine Oxidase (DAO) ELISA Kit	ELISA	RX2D779536
ET	Bovine Endothelin (ET) Quantitative ELISA Kit	ELISA	JRXW774866

 https://doi.org/10.3390/biology15060450
